# Prolonged SARS-CoV-2 infection following rituximab treatment: clinical course and response to therapeutic interventions correlated with quantitative viral cultures and cycle threshold values

**DOI:** 10.1186/s13756-022-01067-1

**Published:** 2022-02-05

**Authors:** Christina S. Thornton, Kevin Huntley, Byron M. Berenger, Michael Bristow, David H. Evans, Kevin Fonseca, Angela Franko, Mark R. Gillrie, Yi-Chan Lin, Marcus Povitz, Mona Shafey, John M. Conly, Alain Tremblay

**Affiliations:** 1 Department of Medicine, Cumming School of Medicine, University of Calgary, Calgary, AB Canada; 2 Alberta Precision Laboratories, Alberta Public Health Laboratory, Calgary, AB Canada; 3 Department of Pathology and Laboratory Medicine, University of Calgary, Calgary, AB Canada; 4 Department of Radiology, University of Calgary, Calgary, AB Canada; 5 Department of Cardiac Sciences, University of Calgary, Calgary, AB Canada; 6 Department of Medical Microbiology and Immunology and Li Ka Shing Institute of Virology, University of Alberta, Calgary, AB Canada; 7 Department of Microbiology, Immunology and Infectious Diseases, University of Calgary, Calgary, AB Canada; 8 Tom Baker Cancer Centre, University of Calgary, Calgary, AB Canada; 9 Foothills Medical Centre, 3300 Hospital Drive NW, Calgary, AB T2N4N1 Canada; 10 Foothills Medical Centre, SSB, 1403 29 St NW, Calgary, AB T2N2T9 Canada

**Keywords:** SARS-CoV-2, Immunocompromised patient, Viral shedding, Therapeutics

## Abstract

**Background:**

Detection of severe acute respiratory syndrome coronavirus 2 (SARS-CoV-2) RNA is completed through reverse transcriptase-PCR (RT-PCR) from either oropharyngeal or nasopharyngeal swabs, critically important for diagnostics but also from an infection control lens. Recent studies have suggested that COVID-19 patients can demonstrate prolonged viral shedding with immunosuppression as a key risk factor.

**Case presentation:**

We present a case of an immunocompromised patient with SARS-CoV-2 infection demonstrating prolonged infectious viral shedding for 189 days with virus cultivability and clinical relapse with an identical strain based on whole genome sequencing, requiring a multi-modal therapeutic approach. We correlated clinical parameters, PCR cycle thresholds and viral culture until eventual resolution.

**Conclusions:**

We successfully demonstrate resolution of viral shedding, administration of COVID-19 vaccination and maintenance of viral clearance. This case highlights implications in the immunosuppressed patient towards infection prevention and control that should consider those with prolonged viral shedding and may require ancillary testing to fully elucidate viral activity. Furthermore, this case raises several stimulating questions around complex COVID-19 patients around the role of steroids, effect of antiviral therapies in absence of B-cells, role for vaccination and the requirement of a multi-modal approach to eventually have successful clearance of the virus.

**Supplementary Information:**

The online version contains supplementary material available at 10.1186/s13756-022-01067-1.

## Background

The coronavirus disease 2019 (COVID-19) pandemic has had a global impact with significant morbidity and mortality. A positive upper respiratory or nasopharyngeal (NP) swab reverse-transcription polymerase chain reaction (RT-PCR) molecular amplification test is commonly used to confirm the presence of severe acute respiratory syndrome coronavirus 2 (SARS-CoV-2) virus. Risk factors for severe disease include older age, medical comorbidities, and immunosuppressed status [1].

Our understanding of COVID-19 has evolved as persistence of SARS-CoV-2 infectious viral shedding and recurrence of PCR positivity after one or more negative samples have been documented in both asymptomatic and symptomatic patients [2]. While RT-PCR is a rapid and easily accessible tool, limitations include prolonged positivity in the absence of infectious virus, thus confounding clinical management around possible re-infection or relapse. Understanding the natural progression of SARS-CoV-2 infectious viral shedding is critical from both an individual’s management but also for public health and hospital infection control. Initial studies suggest infectivity declines within ten days in immunocompetent individuals [3]. Immunocompromised patients (ICPs) represent a unique population whereby prolonged infectious viral shedding and infectivity may occur with disease relapse. We present a complex ICP demonstrating protracted infectious viral shedding for 189 days with three distinct COVID-19 clinical relapses associated with waxing and waning of positive viral cultures and RT-PCR cycle threshold (Ct) values.

## Methods

Nasopharyngeal (NP) swabs were collected at regular intervals beginning shortly after admission and tested by RT-PCR, quantitative culture [4] and whole genome sequencing (Additional file 1: Supplementary Material). The patient provided informed consent with approval of the University of Calgary’s Health Research Ethics Board (no. 20-0444).

## Case presentation

Preceding availability of COVID-19 vaccination, a 58-year-old Caucasian male presented to his primary care provider with one-week of cough, low-grade fever, and general malaise. He was diagnosed with follicular lymphoma 10 months prior and received 6 cycles of chemo-immunotherapy (bendamustine and rituximab), achieving clinical remission, and received an additional dose of maintenance rituximab one month prior. An oropharyngeal swab was positive for SARS-CoV-2, but he did not require hospitalization at that time.

**Fig. 1:**
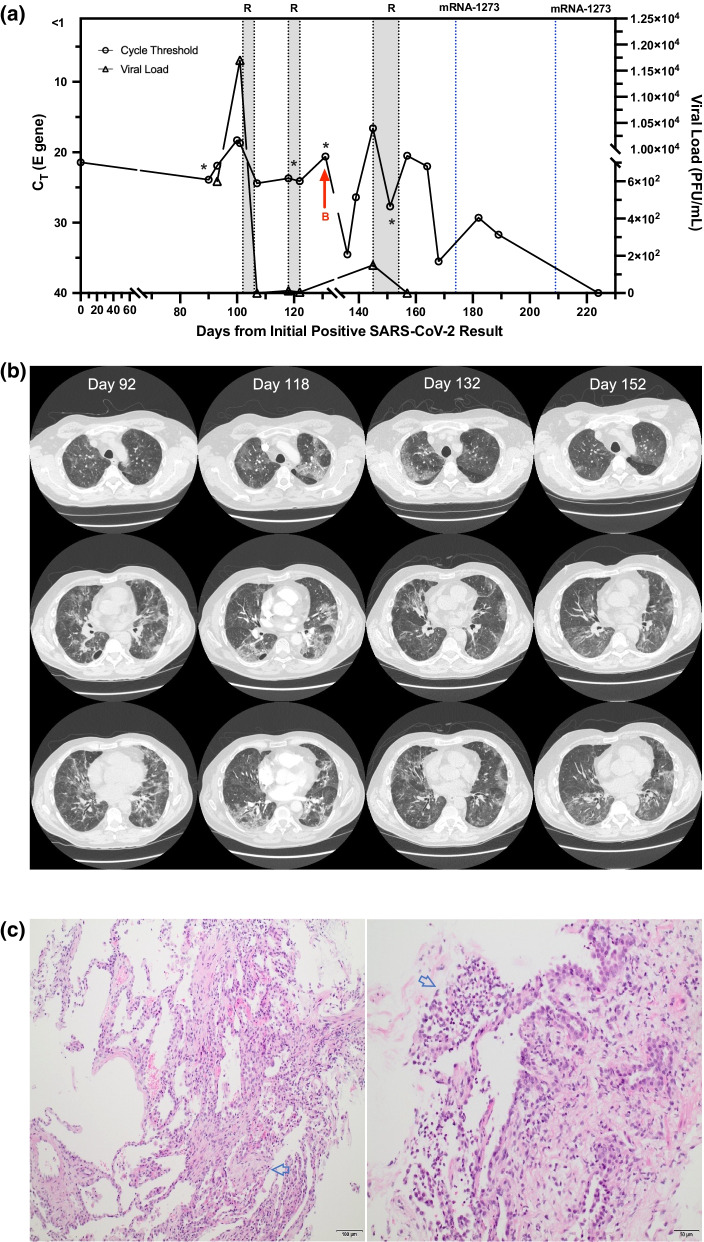
Depiction of prolonged COVID-19 viral shedding in an immunosuppressed patient. **A** Timeline of patient’s clinical course from time of initial COVID-19 positive swab (day 0). Cycle threshold values and culture viral load are represented. Treatment modalities throughout the clinical course are depicted. R: Remdesivir; B: bamlanivimab; *indicates date of corresponding CT scans depicted in B. Cycle threshold values greater than 40 were considered negative. **B** Serial CT scans show multifocal, patchy areas of predominantly ground-glass opacities. The first CT scan shown (day 92) was obtained at the time of the second relapse. The subsequent studies (days 118, 132 and 152) were obtained during the third relapse. On each of the serial scans, some areas of involvement improve while there is worsening in other areas, consistent with migratory opacities. There are features of organizing pneumonia, which is typical later in the course of COVID-19 pneumonia. **C** Transbronchial biopsy. Left image: Low power view of transbronchial biopsy shows alveolated lung parenchyma with expansive interstitial cellular infiltrates composed of lymphocytes and neutrophils. There are subtle occasional foci of organizing pneumonia (blue arrow highlights an intra-alveolar fibroblastic plug of organizing pneumonia) and patchy interstitial fibrosis (hematoxylin–eosin, at original magnification ×10). Right image: High power view with focus of intra-alveolar neutrophils (highlighted with blue arrow) with associated reactive type II pneumocytes lining alveolar spaces (hematoxylin–eosin, at original magnification ×20)

Twenty-seven days after the initial positive swab, he developed hypoxemia prompting hospital admission. Initial NP swab was negative (Fig. 1A) and computed tomography (CT) imaging demonstrated recurrent ground glass opacities (Fig. 1B) with bilateral interstitial pneumonia, compatible with COVID-19. Blood tests revealed lymphopenia (0.1 × 10^9^/L, normal: 0.7–3.5 × 10^9^/L) with increased C-reactive protein (57.0 mg/L, normal: 0.0–8.0 mg/L) and ferritin (2047 μg/L, normal: 30–500ug/L). He improved with dexamethasone 6 mg daily for 10 days. However due to relapsing hypoxemia and radiographic progression, a diagnosis of organizing pneumonia as sequelae of COVID-19 was considered. Bronchoscopy was declined, and oral prednisone 50 mg daily was initiated with a planned dose taper.

On day 90, 73 days post admission, following ongoing reduction of prednisone (10 mg), the patient presented with new onset of pyrexia and recurrence of hypoxemia. RT-PCR testing was again positive by NP swab (Fig. 1A, Ct 18.3). The patient was re-initiated on dexamethasone with bronchoscopy performed. Bronchoalveolar lavage was both SARS-CoV-2 RT-PCR (Ct 18.7) and culture positive (1.17 × 10^4^ pfu/mL, wild-type G clade) and negative for bacterial, mycobacterial, other viral and fungal growth. Transbronchial biopsies (Fig. 1C) demonstrated active pneumonitis with occasional foci of organizing pneumonia, and patchy interstitial fibrosis. Symptoms were consistent with COVID-19 relapse and remdesivir was given (200 mg intravenous loading dose then 100 mg daily) for 5 days concurrently with dexamethasone (Fig. 1A). On day 107, there was clinical improvement with reduction in oxygen requirements to room air and RT-PCR Ct of 24.4 Subsequent investigations revealed hypogammaglobinemia (IgA 0.48 g/L, IgG 3.29 g/L, IgM 0.05 g/L (normal: 0.6–4.2 g/L, 6.8–18.0 g/L, 0.4–3.00 g/L, respectively), likely secondary to prior rituximab administration. He was initiated on monthly intravenous immunoglobulin. He clinically improved and was discharged home on day 107 with an oral dexamethasone (4 mg) taper. Viral culture load at time of discharge was not detectable.

On day 118 the patient re-presented to hospital with respiratory failure. A repeat SARS-CoV-2 RT-PCR swab was positive with Ct of 23.7and a viral culture load of 1.25 × 10^1^ pfu/mL, suggestive of ongoing viral persistence and a third COVID-19 relapse (Fig. 1A). SARS-CoV-2 testing failed to demonstrate remdesivir resistance (supplementary material). He was re-treated with another course of remdesivir for 5 days with modest clinical improvement. Repeat CT scans demonstrated persistent, migratory bilateral ground glass opacities (Fig. 1B). Subsequent medical therapy included bamlanivimab 700 mg (day 133; one dose), IVIG 75 mg (days 137, 165 and 193; one dose each) and a third course of remdesivir (day 146; ten-day course). The patient demonstrated a gradual clinical recovery with progressive reduction in oxygen therapy and eventual negative swab by RT-PCR on day 189. On days 174 and 202, the mRNA-1273 COVID-19 vaccine was administered. On day 202 after initial presentation, he was ultimately discharged home with repeat COVID-19 swabs (days 209 and 224) negative. He has continued to improve with gradually increasing exercise tolerance.

Notably, whole genome sequencing of the patient’s viral samples over the duration of the time-period demonstrated an identical sequence belonging to SARS-CoV-2 clade D614G equivalent to the PANGO B.1 lineage viral strain with a specific sublineage of B.1.160. A review of the local epidemiology during this period noted B.1.160 as the 12th highest lineage, accounting for only 1.4% of the those sequenced from the general population. Based on the sequencing results, relapse of infection by an identical virus strain was demonstrated rather than re-infection with different strains. Finally, serology testing completed was negative to the nucleocapsid antibody, however positive for the spike antibody (value of 1515.3AU/mL; positive threshold of > 50AU/mL).

## Discussion and conclusions

Cases of prolonged infectious viral shedding are emerging in the literature, including B-cell deficiency where shedding of greater than 100 days has been reported [5, 6]. The impact of COVID-19 on immune-deficient individuals and optimal treatment is unknown. Reports of patients post B-cell depletion therapy with spontaneous clearance of SARS-CoV-2 are known; however, others undergoing rituximab therapy had severe and prolonged COVID-19 infections with persistent detectable virus [7].

This case adds to emerging literature on COVID-19 relapse and prolonged SARS-CoV-2 infectious virus shedding in the ICP. Such individuals may pose important infection prevention concerns for similar patient populations as they are often cared in clinical settings with other ICPs and may serve as unrecognized viral reservoirs for superspreading local transmission. Current CDC guidelines recommend duration of isolation for severely immunocompromised COVID-19 patients to be up to 20 days [1]. However, emerging reports have clearly demonstrated prolonged infectious viral shedding far exceeding this timeframe. In the hospital setting, isolation guidelines are of critical importance as prolonged infectious viral shedding poses risk to both patients and healthcare workers.

Emerging data has highlighted the difficulty in SARS-CoV-2 eradication in those with blunted immune responses, prolonged viral shedding, and protracted clinical courses. Rituximab is a chimeric monoclonal antibody that binds to the CD20 molecule on the surface of B-cells leading to B-cell depletion lasting 6–9 months. Theoretically, the CD20 surface molecule is lost during B-cell differentiation into plasma cells, the major source of circulating immunoglobulins, and effects on immunoglobulin production after short courses of rituximab should be a rare event but real-world experience has demonstrated reduced response to vaccines and reactivation of viral infections, such as COVID-19. A recent retrospective study revealed up to a 45% incidence of hypogammaglobinemia in rituximab-treated individuals, persisting up to 42 months, and 4% of patients failing to recover CD20 cellular function [8]. Naïve B-cells, disproportionally depleted by rituximab, are critical to produce neutralizing antibodies in COVID-19 [9]. Similarly, rituximab-treated patients often fail to respond to SARS-CoV2 vaccines, suggesting that B-cells are required not only for viral clearance during natural infection but also for adequate responses to vaccination [9].

Treatments including remdesivir and dexamethasone have shown modest benefit in the general population, but efficacy is unclear in ICPs. The use of COVID-19 convalescent plasma in severely ICPs due to prior treatment with anti-CD20 monoclonal antibodies had early promise with favourable safety efficacy early in disease course, but a recent randomized control trial failed to demonstrate benefit [10]. Similarly, while no strong evidence exists for use of anti-SARS-CoV-2 monoclonal antibody treatment in ICPs, a rational argument for their use in such situations may be made, and in our case resulted in a significant but transient improvement in Ct values. Our study was unique in utilizing a multimodal therapeutic targeted approach and successfully allowing vaccination against COVID-19, while highlighting the profound effect of rituximab on the B cell lineage.

The patient’s clinical course may also have been impacted by corticosteroid treatment. While dexamethasone can improve mortality in severe COVID-19 illness, evidence in immunosuppressed individuals is lacking. The development of organizing pneumonia following COVID-19 illness and associated use of prolonged courses of corticosteroids have been described [11], as highlighted in our case with hypoxemia improving with escalating steroid doses. However, despite initial improvement, additional immunosuppression may have contributed to prolonged viral replication with eventual viral clearance coinciding with tapering of corticosteroid treatment to physiologic doses. Regardless of the mechanisms of viral clearance, all anti-viral therapies attempted provided modest improvement in Ct values and cultivatable virus, although often with transient effect, highlighting the underlying impact of host immune defects.

In conclusion, our case describes a prolonged clinical course with multiple COVID-19 relapses in an immunosuppressed lymphoma patient caused by the same strain of SARS-CoV-2. Our findings have larger implications towards infection prevention and control guidelines that should consider prolonged isolation and rigorous follow-up. Use of quantitative PCR that are well validated and correlated with viral culture titres in parallel with Ct values may serve as a powerful clinical tool to elucidate infectious viral shedding, guide treatments, and assess outcomes. Response to specific anti-viral treatments may be seen, but repeated interventions are likely needed due to relapse while waiting for effects of immunosuppressive agents such as rituximab to diminish. The use of corticosteroids for organizing pneumonia needs to be balanced carefully with the risk of compounding pre-existing immunosuppression.

## Supplementary Information

**Additional file 1**. Viral culture load at time of discharge was not detectable (Fig. 1A).

## Data Availability

Not applicable.

## Abbreviations

SARS-CoV-2: Severe acute respiratory syndrome coronavirus 2
RNA: Ribonucleic acid
RT-PCR: Reverse transcriptase-polymerase chain reaction
COVID-19: Coronavirus disease 2019
ICP: Immunocompromised patients
CT: Computed tomography
IVIG: Intravenous immune globulin
Ct: Cycle threshold
CDC: Centers for disease control and prevention
