# Supplementary material for: Prolonged SARS-CoV-2 infection following rituximab treatment: clinical course and response to therapeutic interventions correlated with quantitative viral cultures and cycle threshold values
Source: Antimicrob Resist Infect Control. 2022 Feb 5;11:28. doi: 10.1186/s13756-022-01067-1 (PMC8817557; doi:10.1186/s13756-022-01067-1)
Supplement: Supplementary file 1 — Additional file 1. Viral culture load at time of discharge was not detectable (Fig. 1A). [file 13756_2022_1067_MOESM1_ESM.docx]

**Supplemental Material**

**Materials and Methods:**

Similar to previously published methods for viral culture^4^, each nasopharyngeal (NP) or oropharyngeal (OP) swab was collected using a polyester tipped NP or throat swab into a sterile container with 3mL universal/viral transport media (Copan UTM-RT (Code 330C or Yocon VTM) or Dulbecco's Modified Eagle’s Medium (Gibco) with addition of 2% fetal bovine serum, 1 µg/ml meropenem and 1µg/ml Amphotericin B (DMEM +). The samples were then placed on ice or refrigerated within 1-2 hours at 4^0^ C and then stored at 4^0^ C for 12- 24 hours at the Public Health Laboratory within Foothills Medical Centre in Calgary (a site within Alberta Precision Laboratories) and transported to the Li Ka Shing Institute of Virology BSL3 laboratories in Edmonton, Alberta, Canada with the authorization of the University of Alberta Human Research Ethics Board (Pro00099761). There, serial 10-fold dilutions of the virus were plated in duplicate on Vero cells (ATCC Cat# CCL-81) and cultured for 3 days at 37˚C in Modified Eagle’s Medium (Gibco) supplemented with 100 units/ml of penicillin, 100 ug/ml of streptomycin and 0.25 ug/ml of Amphotericin B included (Gibco), and 0.9% carboxymethylcellulose (Fisher). The cells were then fixed and stained with formaldehyde and crystal violet to permit plaque counts. Remdesivir resistance testing was completed on viral cultures and defined as viral recovery at a remdesivir concentration of 1.115 μM. The patient sample in this case was recovered at 0.589 μM, indicative of lack of remdesivir resistance. E gene PCR for SARS-CoV-2 was performed as described by Berenger *et al*^12^*.* Any additional samples submitted for diagnostic testing were done using Health Canada/FDA approved tests. Serology blood testing was completed utilizing the Abbott Architect SARS-CoV-2 assays to the nucleocapsid and Spike RBD protein. The full genome of all SARS-CoV-2 identified from this patient were amplified by multiplex PCR using the Freed protocol^13^ as 1,200-bp amplicons or the Resende protocol^14^ and sequenced using Oxford Nanopore or Illumina sequencing technology as previously described^15^.
